# Expression of Concern: The Protective Role of MiR-206 in Regulating Cardiomyocytes Apoptosis Induced by Ischemic Injury by Targeting PTP1B

**DOI:** 10.1042/BSR20191000_EOC

**Published:** 2025-10-08

**Authors:** Yejun Yan, Hongwei Dang, Xin Zhang, Xia Wang, Xiaodong Liu

**Affiliations:** Department of Cardiology, Central Hospital of Baoji City, Shannxi Province 721008, China

*Bioscience Reports* has been made aware of potential issues surrounding the scientific validity of this paper and hence is issuing an Expression of Concern to notify readers whilst the Editorial Office investigates. Concerns have been raised regarding the stylisation and appearance of the Western Blots. The authors have been contacted for comment and as of yet have not responded or provided the requested raw data.

